# Hemostatic parameters in pulmonary tuberculosis patients after intensive phase treatment

**DOI:** 10.22088/cjim.12.3.294

**Published:** 2021-04

**Authors:** Lingga Suryakusumah, Nur Ahmad Tabri, Sahyuddin Saleh, Syakib Bakri, Hasyim Kasim, Andi Fachruddin Benyamin, Erwin Arief, Arifin Seweng

**Affiliations:** 1 Internal Medicine Department, Faculty of Medicine, Hasanuddin University, Makassar, South Sulawesi, Indonesia; 2 Division of Pulmonology and Respiratory, Department of Internal Medicine, Faculty of Medicine, Hasanuddin University, Makassar, South Sulawesi, Indonesia; 3 Division of Hematology and Medical Oncology, Department of Internal Medicine, Faculty of Medicine, Hasanuddin University, Makassar, South Sulawesi, Indonesia; 4 Biostatistics Department, Faculty of Public Health, Hasanuddin University, Makassar, South Sulawesi, Indonesia

**Keywords:** Pulmonary tuberculosis, Hemostatic parameters, Hypercoagulability

## Abstract

**Background:**

Tuberculosis (TB) is an infectious disease caused by mycobacterium tuberculosis (Mtb). This infection causes the release of proinflammatory cytokines that affect hemostasis. Pulmonary TB infection causes an increased activation of procoagulant factors, decreased anticoagulant factors and suppresses fibrinolysis which causes hypercoagulable. Our study is conducted to assess the association between pulmonary TB infection (PTB) with hemostatic parameters before and after intensive phase treatment.

**Methods:**

This was an analytic observational prospective cohort design. The study was conducted at the Community Center for Lung Health in South Sulawesi. Studied subjects were recruited by consecutive sampling, in which the patients who met the inclusion criteria received intensive phase of ATD treatment. PT, aPTT, fibrinogen, and D-dimer were measured before treatment and after the intensive phase of ATD. These data were analyzed using the SPSS Version 22.

**Results:**

In this study, 30 subjects are new cases of PTB. Prothrombin time, aPTT and D-dimer levels were higher in far advanced lesions and smear-positive sputum group (p<0.001). There was a significant level decrease in PT, aPTT, fibrinogen, D-dimer after intensive phase treatment (p<0.001).

**Conclusion:**

Pulmonary tuberculosis infection is associated with hypercoagulability which is characterized by an increase in hemostatic parameters and has significant improvement after intensive phase of ATD treatment.

Tuberculosis (TB), is an infectious disease caused by Mycobacterium tuberculosis (Mtb) and it usually affects lungs, although multiple other organs can also be affected. Tuberculosis also is one of the deadliest infectious diseases worldwide (1). According to a 2015 WHO report, an estimated 9.6 million new TB cases in the world with 3.2 million cases occurred in women, and 1.5 million deaths due to TB (2). Worthy of note, in the science of tuberculosis infection, has been the role of various inflammatory cells, cytokines and immune affectors which mediates in the formation of granulomatous lesions encountered in tuberculosis. In response to this infection, immune cells produce some proinflammatory cytokines such as interleukin (IL)-1, IL-6, and tumor necrosis factor (TNF)-α; affecting homeostasis (1). These changes include increased procoagulant activity (prothrombin time (PT), activated partial thromboplastin time (aPTT), fibrinogen and D-dimer), decreased anticoagulant factors (antithrombin III, protein S, protein C,) and suppress fibrinolysis resulting in hypercoagulable state (3-5).

Currently in Indonesia, there are no data that describe the parameters of hemostasis and the hypercoagulable state in pulmonary TB patients (PTB), therefore our study is conducted to assess the association between pulmonary TB infection with hemostatic parameters before and after intensive phase treatment and it can be considered for prohylaxis treatment with anticoagulant in TB patients with hypercoaguability.

## Methods

**Study population:** The present study was an analytic observational prospective cohort design. The study was conducted at the Community Center for Lung Health in South Sulawesi from July 2019 to September 2019. The inclusion criteria were patients who were diagnosed with new cases of PTB, aged >18 years, willing to take part in the study and signing an inform consent letter. Exclusion criteria were patients with diabetes mellitus, significant liver or autoimmune disorders, use of anticoagulant or immunosuppressive drugs, pregnancy, malignancy, immobilization, and smokers.

**Study design: **Studied subjects were recruited by consecutive sampling, in which the patients who met all of the following inclusion criteria were included in this study until the number of samples was achieved. Diagnosed pulmonary TB was confirmed when at least one of two sputum samples collected tested positive on Ziehl-Neelsen staining or if smear is negative, the diagnosis is established clinically and radiologically (chest x-ray). Patients received antituberculosis drugs (ATDs) treatment for 2 months with a regimen of rifampicin 10 mg/kg, isoniazid 5 mg/kg, pyrazinamide 25 mg/kg and ethambutol 15 mg/kg. 

Prothrombin time, aPTT, fibrinogen, and D-dimer levels were measured in the laboratory before treatment begins. A 1.8 ml of venous blood was drawn in trisodium citrate anticoagulant vial. The citrate blood was centrifuged for 15 minutes at 3000 rpm and plasma was collected in vacutainer tubes. The serum level of PT was measured using Neoplastine^®^ CI Plus 5, while aPTT was measured using STA-C.K PREST^®^ 5 and fibrinogen via Fibri-Prest^® ^Automate 2 (quantitative determination of fibrinogen by Clauss method); all kits from Diagnostica Stago, France; following the manufacturer’s protocol.

Another 3 ml of blood was added into an EDTA container for D-dimer. The sample was done using fluorescence immunoassay device (Triage^®^ D-Dimer Test, Alere, US). The test procedure is carried out by dropping some blood into the port on the device test. Then, it put into the Alere Triage^®^ Meters (referred to as Meter). Samples were analyzed based on the amount of fluorescence detected on the test equipment which will be displayed on the Meter screen in 20 minutes. At the end of second month of ATDs, these hemostatic parameters were repeated.

**Statistical analysis:** The data were analyzed using SPSS Version 22. The statistical analysis conducted was descriptive statistical calculations and frequency distribution as well as paired- t-test, McNemar’s , Fisher’s exact and chi-square tests. The test results are significant if the p-value <0.05.

**Ethical approval:** The study was approved and acknowledged by the Ethics Medical Committee of Hasanuddin University, with reference number: 409/UN4.6.4.5.31/PP36/2019.

## Results

 During the study period, 30 subjects met the inclusion criteria. Table 1 shows that majority of subjects were males (53.3%), aged 18-73 years, had normal nutritional status (86.7%), with positive acid-fast smear sputum and (56.7%) had far advanced lesions on chest x-ray (56.7%).

**Table 1:** Baseline Characteristics (n=30)

**Variabl** **e** **s**		**n**	**%**
Gender	Male	16	53.3
	Female	14	46.7
Age*	<35 years old	15	50.0
	≥35 years old	15	50.0
BMI	Normal	26	86.7
	Obese	4	13.3
AFB Sputum	Positive	17	56.7
	Negative	13	43.3
Lessions	Minimal	13	43.3
	Far Advanced	17	56.7

Note: *Age categories based on Median, BMI: Body mass index, AFB: acid-fast bacilli, n: total

Table 2 demonstrates that PT, aPTT and D-dimer levels before treatment were higher in sputum smear-positive (p<0.001). Fibrinogen levels were higher in AFB smear-positive sputum (615.81±152.61 ng/ ml) compared to smear-negative sputum (511.45±131.48 ng/ml) although statistically not significant (p 0.059). This shows the association between AFB smear-positive sputum with high levels of PT, aPTT, D-dimer.

**Table 2:** Association Beetween AFB Smear Sputum with Hemostatic Parameters Before Treatment

**Parameters**	**AFB ** **Sputum**	**n**	**Mean**	**SD**	**p-value**
PT	Positive	17	15.73	1.62	0.000
	Negative	13	13.19	.63	
aPTT	Positive	17	37.14	2.34	0.000
	Negative	13	31.79	2.41	
Fibrinogen	Positive	17	615.81	152.61	0.059
	Negative	13	511.45	131.48	
D-dimer	Positive	17	1818.18	1438.98	0.000
	Negative	13	182.23	214.31	

Note: PT: prothrombin time, aPTT: activated partial thromboplastin time.

SD: standard deviation, n: total

From table 3 we can see that PT, aPTT and D-dimer levels before treatment is much higher in far advanced lesions than minimal lesions (p<0.001). This illustrates a significant association between the area of chest x-ray lesions with PT, aPTT, and D-dimer levels. Fibrinogen levels were higher in the chest x-ray of far advanced lesions compared to minimal lesions although they were not statistically significant (P= 0.059).

**Table 3:** Association Beetween Chest X-Ray Lesions With Hemostatic Parameters Before Treatment

**Parameters**	**Chest X-Ray Lesions**	**n**	**Mean**	**SD**	**p-value**
PT	Minimal	13	13.19	.63	0.000
	Far Advanced	17	15.73	1.62	
aPTT	Minimal	13	31.79	2.41	0.000
	Far Advanced	17	37.14	2.34	
Fibrinogen	Minimal	13	511.45	131.48	0.059
	Far Advanced	17	615.81	152.61	
D-dimer	Minimal	13	182.23	214.31	0.000
	Far Advanced	17	1818.18	1438.98	

Note: PT: prothrombin time, aPTT: activated partial thromboplastin time,SD: standart deviation, n: total

Table 4 shows significant improvement of PT (n=15, 93.8%), of fibrinogen (n=24, 82.8%), and of D-dimer (n=15, 83.3%) which became normal after treatment (p<0.001). All subjects had normal aPTT after treatment, although it could not be tested statistically.

**Table 4:** Hemostatic parameters and subjects proportion after treatment

Parameters	Improve	Stable	Deteriorate	p-value
PT	15 (93.8%)	15(50%)	0 (0%)	0.000
aPTT*	12 (100%)	18(100%)	0 (0%)	-
Fibrinogen	24 (82.8%)	6 (20%)	0 (0%)	0.000
D-dimer	15 (83.3%)	15 (50%)	0 (0%)	0.000

Note: *aPTT cannot be tested, PT: prothrombin time, SD: standart deviation, aPTT: activated partial thromboplastin time, n: total

Table 5 shows the decrease in hemostatic parameters after treatment of ATD. Prothrombin time levels significantly decreased after treatment from 14.63±1.8 seconds to 12.63±0.7 seconds, aPTT from 34.82±3.56 seconds to 31.15±2.82 seconds, fibrinogen from 570.59±150.89 mg/dl to 341.28±73.09 mg/dl, and D-dimer from 1109.27±1356.94 ng/ml to 242.90±297.90 ng/ml. All of these decreases were statistically significant (p<0.001).

**Table 5:** Comparison analysis for PT, aPTT, fibrinogen,D-dimer in Pulmonary Tuberculosis Patiens

**Parameters**	**Baseline**	**After Intensive phase**	**pvalue**
PT	14.63±1.80	12.64±0.70	0.000
aPTT	34.82±3.56	31.15±2.82	0.000
Fibrinogen	570.59±150.59	341.28±73.09	0.000
D-dimer	1109.27±1356.94	242.40±297.90	0.000

Note: PT: prothrombin time, aPTT: activated partial thromboplastin time.

## Discussion

Tuberculosis infection is characterized immunologically by acute phase responses and hematologically by activation of procoagulation, decreased anticoagulation and disruption of the fibrinolytic system (6,7). This disease can cause thrombosis through various mechanisms such as production of proinflammatory cytokines, venous compression and by local invasion. Previous studies have shown an association between altered hemostatic activity and hypercoagulable state, and it has been proven that hypercoagulable can return to normal after ATD treatment. In this study, we show that PTB is associated with hypercoagulable state as reflected by increased coagulation activity.

 The results show an association between chest x-ray lesions, AFB smear sputum and hemostatic parameters before ATD treatment. Prothrombin time, aPTT and D-dimer levels are higher in far advanced lesions and AFB smear-positive sputum group. Also fibrinogen levels are higher in that group but statistically was not significant. Increased hemostatic parameters are associated with the circulation of pro-inflammatory cytokines in the body. In a cohort study, subjects with AFB smear-positive before treatment had higher levels of IL-6 and interferon-γ compared to AFB smear-negative subjects; IL-6 (=0.035) (8). Moreover in the study of Chowdhury et al., a significant positive correlation was found between the chest x-ray lesions with TNF-α and IL-6 levels (p=<0.001) (9). Our results are in line with the study of Tan et al. which showed a TB with a positive sputum smear results in an increase PT level associated with severe lung injury (10). Immune complexes and many other factors elaborated in various infectious diseases induce IL-1, IL-6, TNF-α production which initiates coagulation activity by increasing stimulation of TF in endothelial cells, monocytes and macrophages, stimulation of intrinsic and extrinsic pathways, as well as inducing hepatic acute phase responses that cause changes in levels of coagulation proteins such as fibrinogen and factor VIII (6,11,12). The PT and aPTT of the PTB patients were prolonged beyond the reference ranges of 10.8-14.4 seconds and 25-36 seconds respectively suggesting that the clotting factors assessed depleted. When a state of chronic inflammation is developed; an integral part of the host defense geared towards eradication of the offending pathogen involves hemostatic system activation as suggested by earlier reports. 

However, an exaggerated systemic activation results in disseminated intravascular coagulation (DIC) with the clotting factors consumed as a consequence. This explains the prolonged PT and APTT observed for TB patients and implies that both extrinsic and intrinsic pathways of blood coagulation are affected (13,14). Fibrinogen production can increase rapidly as a result of a variety of basically non-specific stimuli. Possible risk of deep vein thrombosis (DVT) is significantly increased up to 4 times which is higher in patients with fibrinogen levels >5 g/L (11). Increased fibrin degradation products (FDP) or D-dimers are non-specific indicators in which fibrinolysis has occurred. Fibrin degradation products only measure degraded products while D-dimers are more specific indicators, measuring the activity of thrombin and plasmin by detecting newly formed and newly degraded fibrin (15–17). D-dimers enhance the inflammatory mediators such as IL-1, IL-6, and their levels also increase during process of atherosclerosis. (18). Severe PTB often results in DVT because of the relationship between inflammation and hemostatic changes, causing a hypercoagulable state. However, the occurrence of this hypercoagulable process, depends on several factors such as the level of virulence,and immune response to microorganisms (19). After intensive phase treatment, there was a significant decrease in PT, aPTT, fibrinogen, and D-dimer levels. This result also is in line with the result of Kutiyal et al. which showed a significant decrease in PT and fibrinogen, but despite a decrease in aPTT levels, it was not statistically significant (7). Akpan’s study found a significant decrease in fibrinogen levels after ATD treatment for 2 months followed by 6 months (p<0.001), but the PT and aPTT values were not statistically significant. The purpose of intensive phase treatment is to eliminate *Mycobacteria* and improve symptoms. With the decline in *Mycobacteria* will affect clotting factors that initially depleted to be restored to normal levels in plasma. This may be responsible for the resolution of the prolonged PT and APTT as therapy progressed. The decrease in fibrinogen occurs because the hepatic acute phase response is also suppressed after being treated with ATD, so fibrinogen is no longer produced in large quantities which leads to a decrease in its concentration. Thus, fibrinogen can also be a useful marker in monitoring therapeutic response in TB management (13). 

With anti-TB therapy, there is healing of the endothelium which was damaged by the TB infection. This results in reduced production of plasminogen activator inhibitor as well as inactivation of thrombin-activatable fibrinolysis inhibitor. These inhibitors suppress excessive (secondary) fibrinolytic activity thus normal (primary) fibrinolysis is restored with decreased accumulation of fibrin degradation product and D-dimer as a consequence (20).

 The limitation of this study is that we did not measure anticoagulant levels. However, prolongation of PT and aPTT not only showed depletion of coagulation factors but also illustrated suppression of anticoagulant by proinflammatory cytokines. In conclusion, pulmonary tuberculosis infection is associated with a hypercoagulability which is characterized by an increase in hemostatic parameters which improved significantly after intensive phase treatment.
